# Suicidal Ideation and Suicidal Behaviour in Delusional Disorder: A Clinical Overview

**DOI:** 10.1155/2014/834901

**Published:** 2014-01-30

**Authors:** Alexandre González-Rodríguez, Oriol Molina-Andreu, Víctor Navarro Odriozola, Cristóbal Gastó Ferrer, Rafael Penadés, Rosa Catalán

**Affiliations:** ^1^Department of Psychiatry and Psychology, Hospital Clinic of Barcelona, University of Barcelona, 170 Villarroel Street, 08036 Barcelona, Spain; ^2^Department of Psychiatry, University Hospital Mutua de Terrassa, 5 Doctor Robert Square, 08221 Terrassa, Spain; ^3^Department of Psychiatry and Psychology, Hospital Clinic of Barcelona, University of Barcelona, IDIBAPS, CIBERSAM, 170 Villarroel Street, 08036 Barcelona, Spain

## Abstract

*Background*. Most of the existing studies suggest that suicide is one of the leading causes of premature death in patients with chronic psychotic disorders. However, very few studies have specifically investigated suicidal behaviour in patients with delusional disorder. Thus, our objective was to review the literature regarding the percentage of lifetime ideation and suicidal behaviour in delusional disorder in order to provide suggestions for clinical practice. *Methods*. MEDLINE and PsycINFO were searched from January 1980 to September 2012 using the following keywords: delusional disorder, paranoia, suicidal ideation, and suicidal behaviour. *Results*. A total of 10 studies were identified and included in the review. The percentage of suicidal behaviour in delusional disorder was established between 8 and 21%, which is similar to schizophrenia. Suicidal ideation and suicide attempts were more frequent in patients showing persecutory and somatic delusions in the reviewed studies. *Conclusions*. To the best of our knowledge this is the first attempt to specifically review the suicide phenomenon in patients with delusional disorder. Interestingly, our results support the notion that percentages of both suicidal ideation and behaviour in delusional disorder are similar to patients with schizophrenia.

## 1. Introduction

It is a well-known fact that suicide is a major cause of death in Western countries [1]. Most of the available studies suggest that nearly 70–95% of suicide victims have a mental disorder, the most common being affective disorders and schizophrenia [2]. Some studies have estimated a frequency of suicidal ideation in schizophrenic patients around 50% [2, 3]. In addition, 20–50% of these patients reported suicidal attempts in their lifetime [4].

Regarding this issue, two recent systematic reviews [5, 6] have reported several risk factors that showed a strong association with suicidal behavior, for instance, being young, being a male, having a high level of education, and the presence of comorbid depressive disorders.

When focusing on first episode of psychosis patients, a high risk of suicide has been frequently reported in the first 5 to 10 years of the disease [7].

On the other hand, many studies have also shown that chronic patients with schizophrenia showed high rates of suicide [8]. Given the clinical significance of these findings, many risk factors for suicide have also been investigated in chronic patients with schizophrenia, such as duration of illness, duration of untreated psychosis (DUP), the presence of comorbid depressive symptoms, hopelessness, and previous history of suicide attempts [7, 8].

Despite the paucity of information regarding this topic, a patient-oriented recovery manual focusing on subjective and personal experiences of individuals with schizophrenia and suicidal behaviour has been recently published [9].

In a cross-sectional analysis as part of a prospective study, Montross et al. [3] examined the frequency and correlates of suicidal ideation and past suicide attempts in 132 middle-aged patients with schizophrenia and comorbid subsyndromic depression. Twenty-two percent of the sample reported that life was not worth living and 50% reported suicide attempts once or more during their lifetime. Patients with schizophrenia who had attempted suicide in the past presented higher scores on the Hamilton Rating Scale for Depression (HRSD-17) [10] and higher scores on the General subscale of the Positive and Negative Syndrome Scale (PANSS) [11] than those who did not attempt suicide. Harkavy-Friedman et al. [4] compared demographic and clinical features of 156 patients with schizophrenia and divided the sample into two groups according to the presence or absence of suicidal behaviour. In contrast to previous studies, the authors did not find a higher rate of depression in patients who had attempted suicide than those who had not.

Quite surprisingly, available studies on epidemiological and clinical features of delusional disorder (DD) provide little information about the impact of the suicide phenomenon in these patients. No specific reviews can be found regarding the frequency of suicidal behaviour and suicide risk factors in these patients. Furthermore, the relationship between suicide and other psychiatric symptoms, such as depressive comorbidity or negative and positive symptoms, has not been yet elucidated.

Our hypothesis is that the frequency of suicidal ideation and suicidal behaviour in DD would be similar to those in schizophrenia and associated with specific delusional themes.

We aimed to review the literature that is available for suicidal ideation and suicidal behaviour in DD patients to improve our knowledge about this phenomenon, its psychopathological correlates, and suicide related risk factors.

## 2. Methods

### 2.1. Search Strategy

We searched for studies reporting data on suicidal ideation and suicidal behaviour in patients with DD. We performed electronic searches using MEDLINE and PsycINFO databases from January 1980 to September 2012, by using the following keywords: “delusional disorder,” “paranoia,” “suicidal ideation,” “suicidal behavior,” and “comorbidity.”

Patients met diagnostic criteria for DD according to Diagnostic and Statistic Manual of Mental Disorders DSM-III-TR or DSM-IV criteria. We did not apply restrictions on publication type or design of the study and all relevant papers in English, German, and Spanish were included. In addition, relevant abstracts and references related to the search terms were obtained and examined to identify potential additional studies.

### 2.2. Selection Criteria

We performed a general review of all studies including information about suicidal ideation and suicidal behaviour in DD patients (outpatients or inpatients).

Studies were only included if they met the following criteria: (1) being an original publication in a peer-review journal, (2) studying demographic and clinical variables in DD, and (3) including information regarding suicidal ideation or suicidal behaviour.

### 2.3. Recorded Variables

Suicidal ideation and suicidal behaviour were defined according to the adopted definitions of the Columbia-Suicide Severity Rating Scale (C-SSRS), which is a recommended scale by various international agencies such as the FDA, WHO, JCAHO, and Best Practices Library [12].

Suicidal ideation was defined as the presence of at least one of the following five conditions: (1) wish to be dead; (2) nonspecific active suicidal thoughts; (3) active suicidal ideation with any methods (not plan) without intent to act; (4) active suicidal ideation with some intent to act, without specific plan; or (5) active suicidal ideation with specific plan or intent.

Suicidal behaviour was also defined as a potentially self-injurious act committed with at least some wish to die, as a result of act.

The information extracted from retrieved articles was analysed and described regarding age, gender, psychopathological correlates, and types of DD.

## 3. Results

### 3.1. Identified Studies

In the following, we report our findings by using the keywords previously mentioned and the number of studies that we have included, according to the inclusion criteria.

“Delusional disorder and suicidal ideation”: thirty-one articles can be found, but none have been included because they are focused on body dysmorphic disorder or schizophrenia patients.

“Delusional disorder and suicidal behaviour”: sixty-nine articles were found, one of them focusing on suicide attempts in DD. This one was not included because of the lack of information about rates of suicidal behaviour and suicidal ideation.

“Paranoia and suicidal ideation” and “Paranoia and suicidal behaviour”: forty articles were found, but none was considered in this review due to the lack of inclusion criteria.

“Delusional disorder and comorbidity”: one hundred ninety-six articles were found, of which four fulfilled our inclusion criteria.

We also searched the reference list of the identified articles in the original search for further relevant articles.

A total of 12 articles (all of them clinically descriptive) were included, whereas ten different studies were reported: 3 cross-sectional studies, 1 cross-sectional and longitudinal study, a retrospective study, and 5 case reports. Statistical analysis was not conducted due to the heterogeneity of the assessment instruments that were found to be used in the studies.

The main demographic and clinical characteristics of the reviewed studies are presented in Tables 1 and 2.

### 3.2. Age

Yang and coworkers [17] investigated rates of suicide attempts and risk factors in a sample of 722 admissions to an old age psychiatric unit. DD was the second most common psychiatric diagnosis. Of the 55 attempters included in the study, 11 (20%) were patients with DD. Comorbid depressive and adjustment disorders, substance abuse, methods of attempted suicide, and demographic data were not specified by diagnosis. Nearly half of the sample explained that physical illness was a reason for attempting suicide and depressive symptoms were commonly noted on admission in those who attempted suicide. Unfortunately, these results were not analysed by diagnostic group. In another study including patients with DD, Yassa and Suranyi-Cadotte [24] compared 20 patients that were diagnosed as having late-onset schizophrenia (dementia praecox), 7 patients diagnosed as paraphrenic, and 13 DD patients without hallucinations, according to the 8th and last edition of the Kraepelinian classification [25]. Depressive symptoms as clinical manifestations were not found to differ between the three groups. On the other hand, patients with DD showed no suicide attempts or suicidal thoughts, which contrasts with a 10% rate of suicidal tendencies in patients with late-onset schizophrenia. No statistically significant differences were found. In spite of this finding, DD patients had a higher frequency of physical disorders than the other two groups. Although negative or first-rank symptoms were not reported to be present in patients with DD, validated assessment scales were not used in this study.

### 3.3. Gender Differences

Although gender differences in suicidal behaviour have been extensively studied in many mental disorders, these differences have been poorly studied in DD. A cross-sectional study was conducted in a sample of 86 patients with DD by de Portugal et al. [15]. Fifty-three patients of the sample were female. Suicide risk was evaluated by the mini-international neuropsychiatric interview (MINI) [26], presence and severity of depression, and the Montgomery-Asberg Depression Rating Scale (MADRS) [27] and all the data were analysed according to gender. Males were reported to have higher suicide risk than females (24.2% versus 9.4%), but without reaching statistical significance. PANSS positive and negative subscales were significantly higher in men than in women, as well as depression severity mean scores.

In another study, Wustmann and coworkers [13] evaluated gender-related features in DD as part of the Halle Delusional Syndrome Study (HADES-Study). Sixteen women and seventeen men were followed up. The authors reported that women were more likely to take antidepressant medication in comparison to men. When focusing on suicide attempts, women showed higher rates of suicidal behaviour and lower scores on the positive, negative, and general PANSS subscales than men. Although 55.8% of the patients had depressive symptoms, the authors found no statistically significant gender differences in the presence of depressive symptoms [14].

### 3.4. Psychopathological Correlates

de Portugal et al. [28] carried out a cross-sectional study in 86 outpatients as a phase of the DELIREMP study. The following four psychopathological factors were identified by using a factor analysis of PANSS scores: paranoid, cognitive, schizoid, and affective. These factors were successfully compared with the following external validation markers: comorbid diagnosis of major depressive disorder or dysthymia, MADRS [27] depression scores, and other clinical variables. The affective dimension was associated with somatic delusions, depressive disorders, and a high risk of suicide, being the last variable assessed with the mini-international neuropsychiatric interview (MINI) [26] for DSM-IV disorders.

A previous factor analytic study was performed by Serretti et al. [29] in patients with DD. A four-factor structure was identified by using items of the operational criteria (OPCRIT) for psychotic disorders that were compared with demographic and clinical variables. The authors obtained the following 4 factors: depression, irritability, delusion, and hallucination, but no comparisons were conducted between suicide attempters and the four factors.

### 3.5. Types of DD

When reviewing the influence or relationships of DD type on suicidal ideation and suicide attempts, the following DD types were taken into account: erotomanic, grandiose, jealous, persecutory, somatic, or mixtes types. In brief, DD types are related to the predominant delusional theme, but in case of a lack of the DD themes mentioned above, an unspecified type can be diagnosed.

In the DELIREMP study including a sample of 86 patients with DD, de Portugal and coworkers [16] were the first to describe the presence of comorbid disorders and suicide risk in DD by using a systematic research method. Mini interview [26] for DSM-IV disorders was used for the mentioned assessment. Twenty percent of the total sample had attempted suicide, 15.1% showed suicide risk, and the persecutory type was more likely to present suicide risk and to attempt suicide than other types in terms of rates. However, no statistically significant differences were found. Depressive symptoms, which were evaluated by MADRS [27], were more common in persecutory type patients. Besides, similar scores in the positive and general subscales of PANSS were reported in these patients in comparison to other types, and the grandiose type was more prone to present a low risk of suicide.

A previous study was conducted by Hsiao et al. [18] including 86 outpatients on DD. The authors reviewed retrospectively all medical records and the sample was divided into four groups according to DD types (*persecutory, mixed, jealous, and others*) and according to the presence or absence of depressive symptoms. No statistically significant differences were found between the four groups attending to the presence of depression. The presence or absence of previous suicide history was not investigated by delusional types or patients age. Forty-three percent of the patients reported depressive symptoms at the first psychiatric appointment and 7 of them had prior suicidal attempts. Psychopathological rating scales were not assessed and hence their results could not be used to compare those who attempted suicide and those who had not.

### 3.6. Case Reports

An interesting clinical case of an olfactory reference syndrome was reported by Cruzado et al. [19]. A 35-year-old male perceived an unpleasant odour coming from his feet and he was convinced that this could mortify others. For this reason, the patient attempted suicide by jumping from a second floor. He was diagnosed as having a DD, somatic type (DDST), and received fluoxetine 20 mg q.d. and sulpiride 600 mg q.d. A complete remission was achieved and the suicidal ideation disappeared. Another case was described by Alexander [20] in a patient with DDST who presented a 2-year history of delusional halitosis and two suicide attempts in connection to the delusional belief. Sertraline 200 mg daily was prescribed and the patient presented a complete remission after the increase of the antidepressant therapy. Otani et al. [21] presented a case of a 73-year-old Japanese man who was convinced that he was infested with insects. Due to the polytherapy needed for concomitant somatic illnesses, milnacipran was prescribed. Depressive symptoms and suicidal ideation were displayed during a third recurrence of his DDST and after an increased dosage of milnacipran 50 mg daily, depressive symptoms were resolved. In this context, Dimopoulos and colleagues [22] reported the case of a woman with DDST treated with an aripiprazole-mirtazapine combination. Depressive symptoms were present and the patient reported ideas of death without plan to commit suicide. Furthermore, Shaligram and Choudhury [23] presented a case of a monosymptomatic delusion of poverty (DD unspecified type), whereas the patient presented depressive symptoms and death wishes five years after the onset of the illness. Imipramine 225 mg daily, electroconvulsive therapy, and risperidone 6 mg daily were prescribed, and a complete remission was achieved after 6 months.

## 4. Discussion

To the best of our knowledge this is the first study to specifically review the frequency of suicidal ideation and behaviour in patients with DD. Despite the clinical significance of the suicide phenomenon in psychiatric disorders, there is a surprising lack of data about its impact in this kind of chronic psychotic disorder.

Based on published scientific evidence, we found a frequency of suicidal behaviour in DD of nearly 8–21% in the ten included studies (see Tables 1 and 2).

With regard to suicidal behaviour rates by age, a specific study involving inpatients admitted to a psychogeriatric unit was performed by Yang et al. [17]. About 14% of these patients attempted suicide, but no statistical analysis was performed according to the presence of depressive symptomatology. In contrast to the results of this author, Yassa and Suranyi-Cadotte [24] found no suicide attempts in the group of patients with late-onset DD. In our view, these results must be interpreted with caution, given the completely different diagnostic classifications used by the authors.

Although gender differences in suicidal ideation and behaviour in DD patients have been poorly studied, de Portugal et al. [15] were the first to apply a validated systematic research method that allowed a comparison of suicide attempt rates with psychopathological severity in these patients. The authors reported that about 21% of patients had attempted suicide in their lifetime, which is in line with suicide attempt rates found in schizophrenia (18–55%) [3, 5]. Men had higher suicide risk and higher severity of depression and psychotic symptoms than women. However, no statistically significant differences in suicide risk were found in terms of gender. By contrast, Montgomery and Asberg [27] reported a higher rate of suicide attempts in women and lower scores on the PANSS scale, positive, negative, and general PANSS subscales than men. No statistically significant gender differences were found on depressive comorbidity. Therefore, results regarding gender differences on suicidal behaviour in DD are inconclusive, and severity of depressive symptoms in patients with DD could not even be associated with a high suicide rate when investigating by gender.

Suicidal thoughts and suicide attempts would be more frequent in patients with DD persecutory type and DDST, which are the most common delusional ideas encountered in many studies [14, 15, 30, 31]. These findings were described in the DELIREMP study [15] that reported higher rates of suicide attempts and a higher severity of depressive symptoms in patients with persecutory DD than in other types.

Despite the heterogeneity of symptoms in DD, current classifications divide it into seven types according to the content of delusions. However, there is little empirical evidence about the course and prognosis of the different types [32, 33]. Hsiao and coworkers [18] reported seven suicide attempts in patients presenting depressive symptoms at the first psychiatric appointment.

Five case reports have described the presence of suicidal ideation and/or suicidal behaviour in DD. Four out of 5 had DDST, whereas 2 had attempted suicide. All patients received antidepressant treatment alone or in combination with antipsychotics.

de Portugal et al. [28] identified four factors which were associated with different clinical variables. The authors mentioned above concluded that the affective factor, above the rest, was directly related to an increased risk of suicide and a higher frequency of somatic delusions. Therefore, it can be concluded that the current classification based on delusional content alone has not allowed an accurate prognosis of these patients.

Prospective longitudinal studies are needed to better clarify the relationship between the clinical and demographic variables and suicidal behaviour in DD. Furthermore, it is necessary to analyse the available studies on DD according to the presence or absence of suicidal ideation and suicide attempts and to investigate the role of antidepressants in prevention and treatment strategies of psychotic patients with risk of suicide, as it has been reported in schizophrenia patients in the elderly [34]. Specific studies evaluating the effectiveness of antidepressants on depressive symptoms and suicidal risk in DD are needed.

As it has been described in schizophrenia, preventive measures and programs on suicide and other integrated strategies should be implemented when treating DD patients with risk of suicide [35].

We hope that this review will stimulate interest in the study of suicidal behaviour in DD and encourage further research on this topic.

## 5. Limitations 

Due to the qualitative nature of this review and the lack of information regarding this topic, we found only 10 studies suitable for inclusion. The results of this clinical review have indicated some of the methodological problems of the studies conducted to date, such as the heterogeneity of psychopathological tools used in the assessment of suicidal behaviour and suicidal ideation in DD patients. For this reason, we cannot consistently associate suicidal behaviour with affective or psychotic symptoms.

## Figures and Tables

**Table 1:** Summary of prospective, retrospective, and cross-sectional studies on suicidal behaviour in delusional disorder.

(Number of study) author and year	Setting	Country	Study design	Sample size, DD (*n*)	Sex (female/male)	Mean age (SD)	Assessment tools (depression, suicide)	Suicidal ideation *n* (%)	Suicide attempts *n* (%)	Suicide risk *n* (%)	Presence of depression *n* (%)	Treatment on antidepressants *n* (%)
(1) Wustmann et al. 2011 [13]	Inpatient	Germany	Cross-sectional and longitudinal	43	21/22	46.9 (13.2)	BPRS, AMDP system	NA	7 (21.2)	NA	24 (55.8)	17 (39.5)
(1) Wustmann et al. 2012 [14]	Inpatient	Germany	Cross-sectional and longitudinal	43	21/22	46.9 (13.2)	BPRS, AMDP system	NA	7 (21.2)	NA	24 (55.8)	17 (39.5)
(2) de Portugal et al. 2010 [15]	Outpatient	Spain	Cross-sectional	86	53/33	54 (14.4)	MADRS, MINI, PANSS	NA	NA	13 (15.1)	41 (47.7)	35 (40.7)
(2) de Portugal et al. 2009 [16]	Outpatient	Spain	Cross-sectional	86	53/33	54 (14.4)	MADRS, MINI, PANSS	NA	118 (20.9)	13 (15.1)	41 (47.7)	NA
(3) Yang et al. 2001 [17]	Inpatient	China	Cross-sectional	80	NA	NA (Elderly)	Inpatient records review	NA	11 (13.75)	NA	NA	NA
(4) Hsiao et al. 1999 [18]	Outpatient	China	Retrospective	86	42/44	42.4 (15.41)	Medical record review	NA	7 (8.14)	NA	37 (43)	NA
(5) Yang et al. 2001 [17]	Inpatient	China	Cross-sectional	13	12/1	71.3 (9.0)	Interview	NA	0 (0)	NA	3 (23)	0

DD: delusional disorder, SD: standard deviation, BPRS: Brief Psychiatric Rating Scale, MINI: mini-international neuropsychiatric interview, NA: not available, and MADRS: Montgomery Asberg Depression Rating Scale.

**Table 2:** Summary of case reports on suicidal behaviour in delusional disorder.

(Number of study) author and year	Setting	Country	Age (years)	Sex	DD type	Suicidal ideation (yes/no)	Suicide attempts (yes/no)	Presence of depression (yes/no)	Treatment on antidepressants (mg/day)	Treatment on antipsychotic (mg/day)
(6) Cruzado et al. 2012 [19]	Inpatient	Peru	35	Male	Somatic (olfactory reference syndrome)	Yes	Yes (self-defenestration from a second floor)	No	Fluoxetine (20)	Sulpiride (600)
(7) Alexander 2010 [20]	Outpatient	Australia	29	Male	Somatic (delusional halitosis)	Yes	Yes (two suicide attempts)	No	Sertraline (200)	No
(8) Otani et al. 2010 [21]	Outpatient	Japan	73	Male	Somatic (infestation)	Yes	No	Yes	Milnacipran (50)	No
(9) Dimopoulos et al. 2008 [22]	Outpatient	Greece	51	Female	Somatic (hypochondriac)	Yes (ideas of death, not plan to commit suicide)	No	Yes	Mirtazapine (90)	Aripiprazole (15)
(10) Shaligram and Choudhury 2006 [23]	Outpatient	India/UK	48	Female	Not specified (delusion of poverty)	Yes (death wishes)	No	Yes	Imipramine (225) + ECT	Risperidone (6)

DD: delusional disorder, ECT: electroconvulsive therapy.
